# BPIFB4 Circulating Levels and Its Prognostic Relevance in COVID-19

**DOI:** 10.1093/gerona/glab208

**Published:** 2021-08-16

**Authors:** Elena Ciaglia, Valentina Lopardo, Francesco Montella, Carmine Sellitto, Valentina Manzo, Emanuela De Bellis, Teresa Iannaccone, Gianluigi Franci, Carla Zannella, Pasquale Pagliano, Paola Di Pietro, Albino Carrizzo, Carmine Vecchione, Valeria Conti, Amelia Filippelli, Annibale Alessandro Puca

**Affiliations:** 1 Department of Medicine, Surgery and Dentistry “Scuola Medica Salernitana,” University of Salerno, Baronissi, Italy; 2 Department of Clinical Pharmacology and Pharmacogenetics Unit, University Hospital “San Giovanni di Dio e Ruggi d’Aragona,”Salerno, Italy; 3 Department of Experimental Medicine, University of Campania “Luigi Vanvitelli,”Naples, Italy; 4 Infectious Diseases Unit, University Hospital “San Giovanni di Dio e Ruggi d’Aragona,”Salerno, Italy; 5 Department of AngioCardioNeurology, IRCCS Neuromed, Pozzilli, Isernia, Italy; 6 Cardiovascular Research Unit, IRCCS MultiMedica, Milan, Italy

**Keywords:** Immunity function, Longevity, Plasma, SARS-CoV-2

## Abstract

Aging and comorbidities make individuals at greatest risk of COVID-19 serious illness and mortality due to senescence-related events and deleterious inflammation. Long-living individuals (LLIs) are less susceptible to inflammation and develop more resiliency to COVID-19. As demonstrated, LLIs are characterized by high circulating levels of BPIFB4, a protein involved in homeostatic response to inflammatory stimuli. Also, LLIs show enrichment of homozygous genotype for the minor alleles of a 4 missense single-nucleotide polymorphism haplotype (longevity-associated variant [LAV]) in BPIFB4, able to counteract progression of diseases in animal models. Thus, the present study was designed to assess the presence and significance of BPIFB4 level in COVID-19 patients and the potential therapeutic use of LAV-BPIFB4 in fighting COVID-19. BPIFB4 plasma concentration was found significantly higher in LLIs compared to old healthy controls while it significantly decreased in 64 COVID-19 patients. Further, the drop in BPIFB4 values correlated with disease severity. Accordingly to the LAV-BPIFB4 immunomodulatory role, while lysates of SARS-CoV-2-infected cells induced an inflammatory response in healthy peripheral blood mononuclear cells in vitro, the co-treatment with recombinant protein (rh) LAV-BPIFB4 resulted in a protective and self-limiting reaction, culminating in the downregulation of CD69 activating-marker for T cells (both TCD4+ and TCD8+) and in MCP-1 reduction. On the contrary, rhLAV-BPIFB4 induced a rapid increase in IL-18 and IL-1b levels, shown largely protective during the early stages of the virus infection. This evidence, along with the ability of rhLAV-BPIFB4 to counteract the cytotoxicity induced by SARS-CoV-2 lysate in selected target cell lines, corroborates BPIFB4 prognostic value and open new therapeutic possibilities in more vulnerable people.

Severe acute respiratory syndrome coronavirus 2 (SARS-CoV-2) causing COVID-19 pandemic is now well known to be a Coronaviridae family virus which spreads quickly in the whole human population through direct contact and aerosol transmission among people in close contact.

The wide-ranging symptoms have made the understanding of the epidemiological potential of COVID-19 very challenging for the scientific community. COVID-19 patients can be categorized as asymptomatic, mild/moderate, or severe cases. Acute respiratory distress syndrome and multiple-organ failure characterize high severe COVID-19 cases in addition to cough, fever, fatigue, myalgia, dyspnea, and bilateral lung infiltration that are also peculiar of milder forms of the disease (1,2). In addition, disseminated intravascular coagulation, leading to acute cardiac injury and pulmonary embolism, is commonly observed in severe cases and is strongly associated with mortality (2,3).

A growing number of data highlighted that SARS-CoV-2 infection overly boosts the immune system leading to a “cytokine storm” (4,5). After a proteolytic processing of the spike protein, SARS-CoV-2 binds ACE2 receptor expressed in host nasal epithelial cells, lungs, and bronchial branches. Following the membrane fusion and viral endocytosis, SARS-CoV-2 starts to replicate in the host cells and triggers an uncontrolled immune response associated with a defective virus clearance and an inflammatory imbalance. The high-level activation of immune cells results in a high production of inflammatory cytokines (ie, IL-6, IL-2R, IL-7, IL-8/CXCL8, IP10, MCP-1/CCL2, MIP1A/CCL3, TNF-α) that encounters a lack of anti-inflammatory pattern required to fine tune the cytokine-driven response, eliciting acute lung damage and fatal multiple-organ failure (6).

Host co-factors (ie, old age, comorbid chronic conditions) hold the key to establish the severity of COVID-19 infection outcome. It is not surprising that old people are the most vulnerable to COVID-19 seen that aging is notoriously related to a *low-grade* chronic inflammation (ie, inflammaging) (7) that drives a frailty condition, which may become breeding ground for the COVID-19 onset (8,9). In support, a recent meta-analysis highlighted that the increase in clinical frailty score was positively associated with the increase of mortality outcome in old patients with COVID-19 (10).

Studies in long-living individuals (LLIs) clearly indicated that, in spite of their extreme chronological age, they are protected from and cope better with age-related diseases, mainly cardiovascular ones (11). This led to hypothesize that LLIs may show a better response to environmental challenges, such as SARS-CoV-2 infection (9). Accordingly, a degree of resiliency of male centenarians has been recently registered in the Lombardy region, Italy (12).

In this scenario, the immune asset and circulating soluble factors that characterize LLIs may offer new disease biomarkers and therapeutic opportunities in COVID-19 pandemic.

The bactericidal/permeability-increasing fold-containing family-B-member-4 (BPIFB4) protein was found being particularly abundant in respiratory secretion, upper airways and proximal trachea (13,14). Noteworthy, elevated level of this innate immunity belonging protein also selectively marks the plasma of healthy LLIs compared to frail ones (15,16). This allowed us to speculate a protective role against cardiovascular effects of aging. Indeed a longevity-associated variant of the BPIFB4 (LAV-BPIFB4) demonstrated a pleiotropic activity to maintain cellular homeostasis and vessel endothelium integrity along with a surprising capability to orchestrate a balanced immune response (17). Regarding the latter, LAV-BPIFB4 drives a protective macrophage-polarizing effect toward a pro-resolving M2 phenotype coupled with an improvement of the inflammatory arm (ie, reduced IL-1β and TNF-α levels and increased IL-33 levels) (18) and redirects peripheral monocyte differentiation into regulatory dendritic cells that can counteract the low-grade chronic inflammatory condition (14).

Based on these premises, we hypothesized a role of BPIFB4 in mitigating the inflammatory imbalance and the cytotoxic effect of SARS-CoV-2 and the related COVID-19 infection severity.

To investigate this putative effect, COVID-19 patients consecutive admitted at the Infectious Diseases Unit of the University Hospital of Salerno have been recruited and layered into *high-grade* or *low-grade* disease patients based on oxygen saturation values with the needing of oxygen or intensive care unit (ICU) admission and C-reactive protein (CRP) and lactate dehydrogenase (LDH) levels.

To evaluate a potential correlation between BPIFB4 and the severity status of the disease, we detected and quantified the plasma levels of circulating BPIFB4. Then, we stimulated, in vitro, peripheral blood mononuclear cells (PBMCs) and/or a panel of target cell lines (BEAS-2B bronchial epithelial cells, A549 alveolar basal epithelial cells, and human umbilical vein endothelial cells [HUVEC]) with inactivated SARS-CoV-2 lysate. Ultimately, we performed a LDH assay and phenotypical and functional immune assays to evaluate the recombinant LAV-BPIFB4 (rhLAV-BPIFB4) ability in counteracting the lysate-induced cytotoxicity and counterbalancing the pro-inflammatory reaction following the infection.

## Method

### Patient Recruitment

A cohort of 171 individuals has been recruited to perform the study: *n* = 49 LLIs (age > 95; median age 96, range 95–99) constituting the control group for the BPIFB4 levels dosage; *n* = 58 SARS-CoV-2-negative individuals (median age 64, range 24–81); *n* = 64 SARS-CoV-2-positive individuals (median age 65, range 20–91). For each individual, detailed anamnesis and plasma from venous blood was collected for the analyses. All participants signed an informed consent for the management of personal anamnestic data and blood samples. The SARS-CoV-2-positive group had received a diagnosis of COVID-19 based on a positive naso-pharyngeal swab for SARS-CoV-2 RNA. Peripheral blood samples were collected from each patient within 7 days from the admission to the Infectious Diseases Unit of “Giovanni di Dio e Ruggi d’Aragona” University Hospital of Salerno. Clinical laboratory analyses testing at hospital admission included the following: complete blood count (leucocytes, lymphocytes, platelets), mean corpuscular volume, hematocrit, hemoglobin, erythrocyte sedimentation rate, LDH, serum ferritin, D-dimer, CRP, and fibrinogen (Supplementary Table 1). Patients were stratified in 2 groups according to needing of oxygen or ICU admission. Thirty-two cases (*low grade*) with oxygen saturation between 90% and 94% that did not need ICU admission were considered as having mild–moderate COVID-19. Thirty-two patients (*high grade*) with an oxygen saturation below 90% at admission or during the hospital stay that required either noninvasive or mechanical ventilation or need of admission to the ICU were considered as having severe COVID-19.

The study was approved by the IRCCS MultiMedica and by the internal Ethics Committee of “Giovanni di Dio e Ruggi d’Aragona” University Hospital of Salerno and conducted in accordance with the ethical principles deriving from the Declaration of Helsinki.

### Cell Lines and Culture Condition

Adenocarcinomic human alveolar basal epithelial cells (A549 cells, ATCC CCL-185) were grown in a humidified incubator at 37°C and 5% CO_2_ in DMEM (Gibco, Thermo Fisher Scientific) supplemented with 10% (v/v) fetal serum bovine (FBS, Gibco, Thermo Fisher Scientific), 1% (v/v) penicillin–streptomycin (Aurogene), 1% (v/v) MEM non-essential amino acids (MEM NEAA, Gibco, Thermo Fisher Scientific), and 1% (v/v) sodium pyruvate (Aurogene).

Human bronchial epithelial cells (BEAS-2B, ATCC CRL-9609) were grown in a humidified incubator at 37°C, 5% CO_2_ in DMEM/F12 (Lonza BioWhittaker) supplemented with 15% FBS (Gibco, Thermo Fisher Scientific), 1% (v/v) penicillin–streptomycin (Aurogene), and 0.35% (w/v) glucose.

HUVECs (ATCC CRL-1730) were grown in a humidified incubator at 37°C and 5% CO_2_ in Vascular Cell Basal Medium supplemented with Endothelial Cell Growth Kit (ATCC PCS100030). Commercially available HUVECs were obtained from ATCC.

*Cercopithecus aethiops* epithelial kidney cells (Vero cells, ATCC CCL-1586) were grown in a humidified incubator at 37°C and 5% CO_2_ in Eagle’s Minimal Essential Medium (Microgem) supplemented with 10% (v/v) FBS (Microgem), 1% (v/v) L-glutamine (Lonza), 1% (v/v) sodium pyruvate (Lonza), and 1% (v/v) penicillin–streptomycin (Lonza).

PBMCs were extracted from whole blood of healthy donor by Ficoll density gradient (Histopaque-1077, Sigma–Aldrich). After separation, PBMCs were washed and collected in RPMI-free (Gibco, Thermo Fisher Scientific) or RPMI (Gibco, Thermo Fisher Scientific) supplemented with 10% (v/v) FBS (Gibco, Thermo Fisher Scientific), 1% (v/v) penicillin–streptomycin (Aurogene), 1% (v/v) MEM non-essential amino acids (MEM NEAA, Gibco, Thermo Fisher Scientific), and 1% (v/v) sodium pyruvate (Aurogene) for the subsequent experiments.

For the generation of peripheral blood lymphocytes, CD14+ monocytes were depleted from PBMCs by immunomagnetic procedure (MiltenyBiotec).

HUVECs, A549, and BEAS-2B cells were pretreated with recombinant LAV-BPIFB4 (18 ng/mL) for 2 hours and then treated with inactivated SARS-CoV-2 lysate (50 μg/mL) for 72 hours. Following 72 hours of treatment, all supernatants were collected for further analysis. As regards as PBMCs, after the canonical 2 hours of recombinant LAV-BPIFB4 (18 ng/mL) pretreatment, PBMCs were exposed to inactivated SARS-CoV-2 lysate (50 μg/mL) for the following 2 hours. Following this time, in order to avoid the “activation-induced cell death” (AICD) in vitro, RPMI-FBS medium (with or without SARS-CoV-2 lysate) was discarded and fresh RPMI-FBS medium with rhLAV-BPIFB4 (18 ng/mL) was added in order to culture the cells for additional 46 hours or 70 hours. At the end of the culture, supernatants were collected and cells were assayed by flow cytometry.

### SARS-CoV-2 Lysate Production

SARS-CoV-2 lysate was propagated in Vero cells and produced in the presence of 0.5% Triton X 100 plus 0.6 M KCl. After that, it was inactivated at 95°C for 5 minutes. All experimental work involving SARS-CoV-2 was performed in a biosafety level 3 (BSL3) containment laboratory. SARS-CoV-2 clinical isolate was kindly donated by Lazzaro Spallanzani Hospital, Rome, Italy.

### Cytotoxicity Assay

SARS-CoV-2-induced cytotoxicity was determined using commercially available kit (Cytotoxicity Detection KIT LDH, Roche) according to the manufacturer’s instructions. One hundred microliters of reaction mix was added to 100 μL of BEAS-2B, A549, or HUVEC conditioned media collected as above mentioned. Absorbance at 490 nm was measured using a microplate reader.

### Enzyme-Linked Immunosorbent Assay

BPIFB4 plasma levels were determined using Human Long palate, lung, and nasal epithelium carcinoma-associated protein 4 (C20orf186) ELISA kit (Cusabio CSBYP003694HU) following the manufacturer’s protocol. Briefly, plasma was incubated for 2 hours at 37°C in the assay-coated microplate. After removing any unbound substances, a biotin-conjugated antibody specific for C20orf186 was added to the wells and incubated for 1 hour at 37°C. After washing, avidin-conjugated horseradish peroxidase (HRP) was added to the wells and incubated for 1 hour at 37°C. Following a wash, substrate solution was added and the consequent color development was stopped. Optical density was measured at 450 nm.

### Cytokines Detection

Cytokines levels in PBMCs-conditioned media, BEAS-2B, and HUVECs supernatants were determined using a beads-based multiplex ELISA (LEGENDplex, Biolegend, USA). Conditioned media were incubated for 2 hours with the beads and for 1 hour with the detection antibodies, followed by 30 minutes incubation with SA-PE. After washing, beads were resuspended in washing buffer and acquired using a FACS VERSE flow cytometer (BD Biosciences). Data were analyzed with the LEGENDplex Data Analysis Software.

### Antibodies and Flow Cytometry

PBMCs (treated as above mentioned) were stained with mAb against human CD3 PerCP-Cy5.5 (Biolegend #300327, 5 μL/test) and CD69 FITC (Miltenyi Biotec, 5 μL/test) CD4 PE-Vio770 (Miltenyi Biotec, 2 μL/test), and CD8 APC-Vio770 (Miltenyi Biotec, 2 μL/test). After 30 minutes incubation at 4°C in the dark, cells were washed, centrifuged, and resuspended in staining buffer for the FACS analysis. For each test, cells were analyzed using FACS Verse Flow Cytometer (BD Biosciences).

### Statistical Analysis

In all experiments shown, statistical analysis was performed by using the GraphPad prism 6.0 software for Windows (GraphPad software). For each type of assay or phenotypic analysis, data obtained from multiple experiments are calculated as mean ± *SD* and analyzed for statistical significance using appropriate tests. In analysis of variance (ANOVA) for multiple comparison, *p* values < .05 were considered significant; *p < .05, **p < .01, and ***p < .001.

## Results

### BPIFB4 Blood Levels Are Decreased in COVID-19 Patients

BPIFB4 has been shown to serve as a biomarker of healthy aging (15,16) and previous finding on its prognostic significance in vascular pathology (ie, atherosclerotic patients) (18), prompted us to examine BPIFB4 contribution in COVID-19.

**Figure 1.**
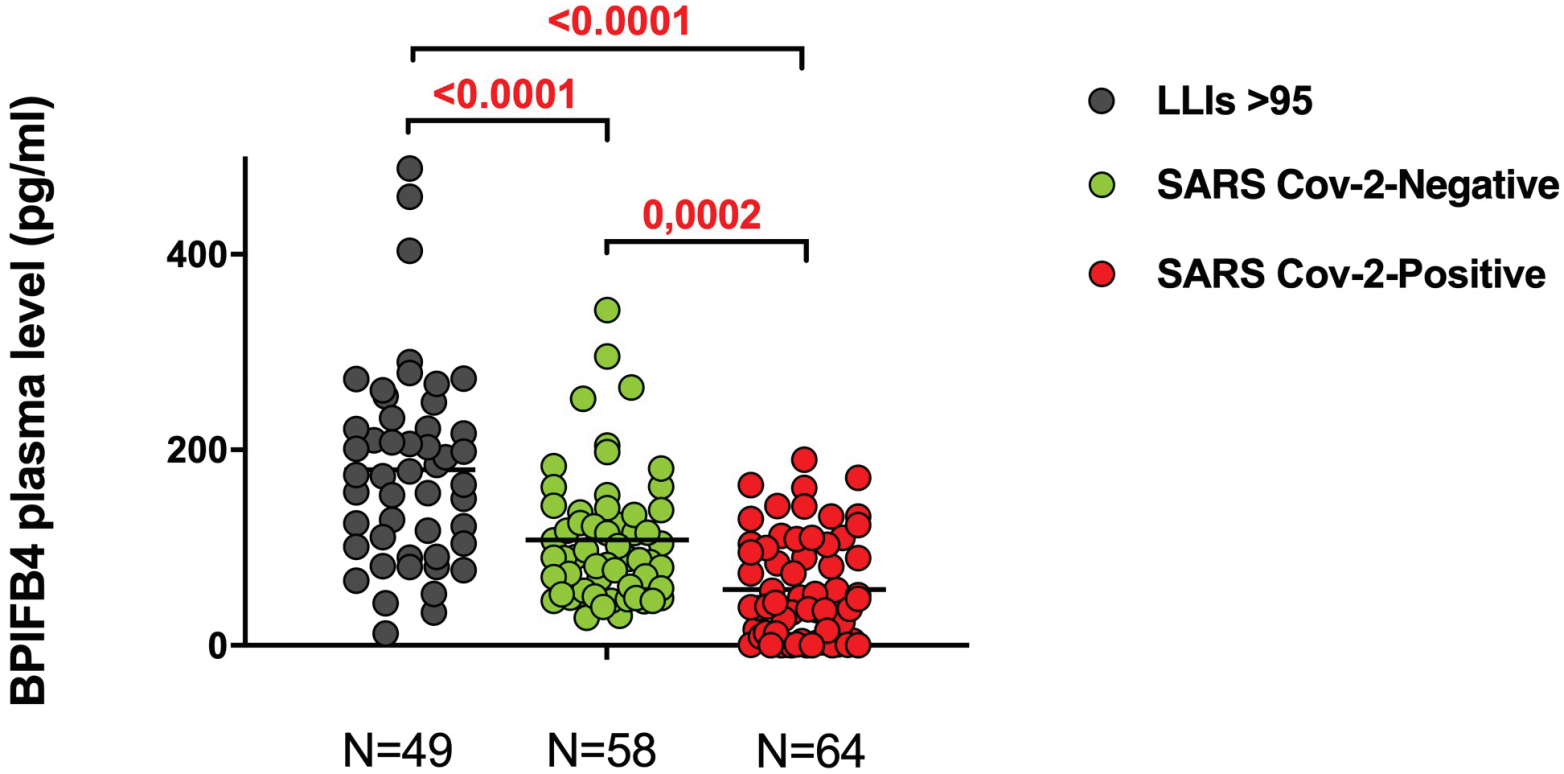
Circulating levels of BPIFB4 protein from long-living individuals (LLIs), SARS CoV-2-negative and SARS CoV-2-positive subjects. ELISA quantification of BPIFB4 levels in plasma from *n* = 49 LLIs (median age 96, range 95–99), *n* = 58 SARS CoV-2 negative (median age 55, range 42–62) and *n* = 64 SARS CoV-2-positive subjects (median age 65, range 20–91) expressed in mean ± *SD*. Statistical evaluation was carried out with 1-way analysis of variance corrected for multiple comparisons by false discovery rate using 2-stage linear step-up procedure of Benjamini, Krieger, and Yekutieli (GraphPad Prism). The individual *p* values are shown.

We examined the plasma BPIFB4 levels in *n* = 64 patients with COVID-19 (median age 65, range 20–91) consecutively admitted to “San Giovanni di Dio e Ruggi d’Aragona” University Hospital of Salerno. Clinical and laboratory features of COVID-19 patients are shown in Supplementary Table 1. For comparison, both a first cohort of *n* = 49 LLIs (age > 95; median age 96, range 95–99) and a second cohort of *n* = 58 SARS-CoV-2-negative individuals (median age 64, range 24–81) constituted the 2 control groups for the BPIFB4 levels dosage (Figure 1).

Importantly, BPIFB4 values were significantly lower in SARS-CoV-2-positive individuals as compared with SARS-CoV-2-negative ones (57.31 ± 53.13 pg /mL vs 108.1 ± 66.4 pg /mL, *p* = .0002) pointing to BPIFB4 as a *bona fide* biomarker inversely related to COVID-19 diagnosis. In parallel, we confirmed that LLIs have higher levels of BPIFB4 as compared to old healthy controls (179.80 pg/mL ± 100.4 vs 108.1 ± 66.4 pg/mL; *p* < .0001; Figure 1).

**Figure 2.**
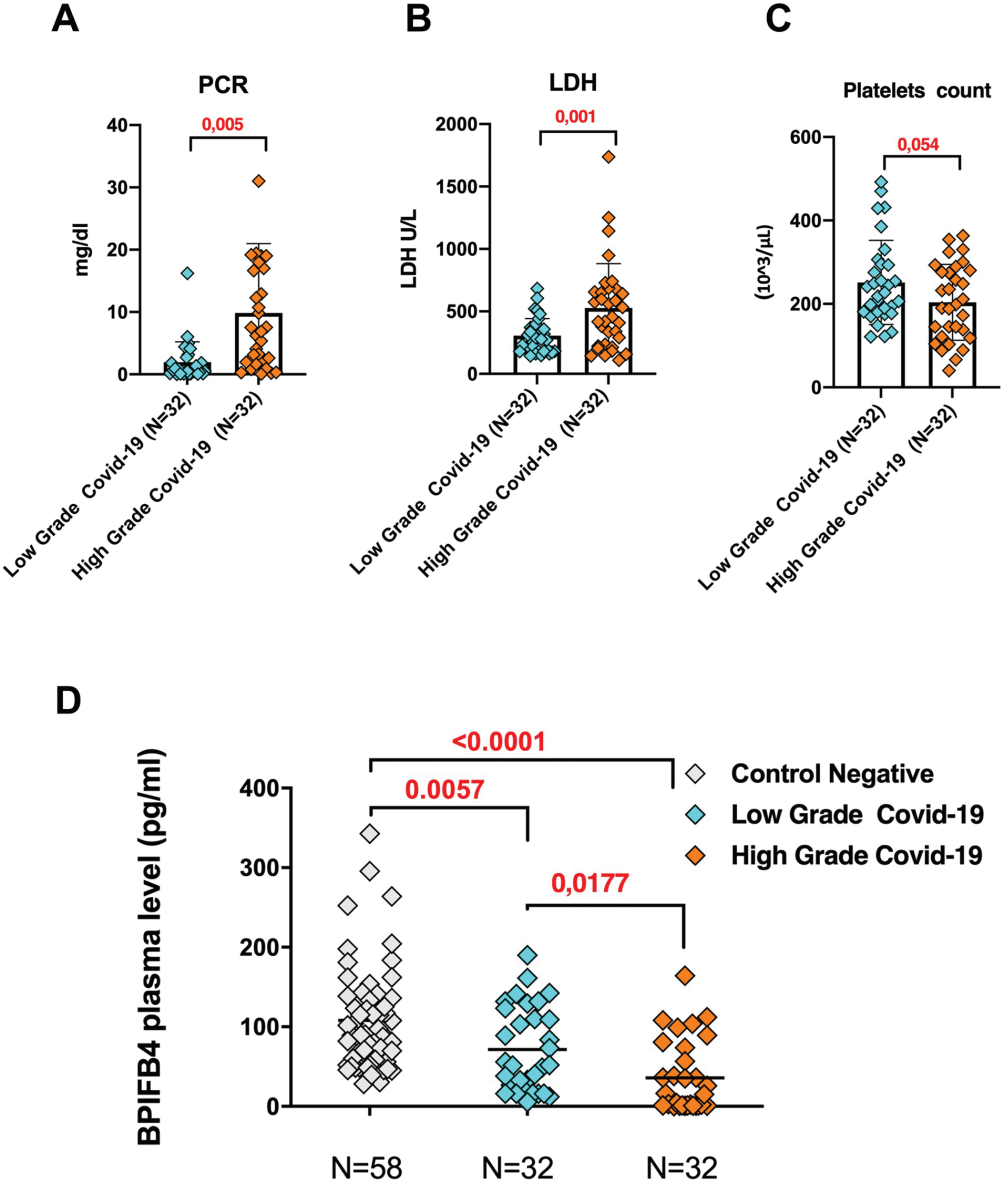
BPIFB4 levels are reduced in severe SARS-CoV-2 patients. Significant differences in laboratory levels of C-reactive protein, lactate dehydrogenase, and platelets count in *n* = 32 severe (high grade) (median age 62, range 88–32) and *n* = 32 nonsevere (low grade) (median age 68, range 20–91) COVID-19 patients (**A–C**). ELISA quantification of BPIFB4 levels in plasma from the 2 groups of SARS-CoV-2 positive patients (high grade and low grade) compared with the group of *n* = 58 SARS-CoV-2-negative patients (**D**). Statistical evaluation was carried out with 1-way analysis of variance corrected for multiple comparisons by false discovery rate using 2-stage linear step-up procedure of Benjamini, Krieger, and Yekutieli (GraphPad Prism). The individual *p* values are shown.

Based on previous studies on the prognostic relevance of BPIFB4 in vascular pathology and its association with the degree of carotid stenosis in atherosclerotic patients (18), we moved to analyze the plasma BPIFB4 levels in *n* = 32 severe (high grade) and *n* = 32 nonsevere (low grade) COVID-19 patients. In our cohort of *n* = 64 COVID-19 subjects, the high-grade patients presented with oxygen saturation values <90% and needing of oxygen or ICU admission. On the basis of the laboratory test, as previously reported (19,20), we observed higher significant levels in LDH and CRP and reduced platelets count in severe patients with respect to nonsevere ones, indicating inflammatory burst in high-grade group (Figure 2A and B). Of note, the average plasma BPIFB4 level in the severe group (high grade) was significantly lower than in nonsevere group (low grade) (35.91± 45.22 pg /mL vs 71.54 ± 52.84 pg /mL, *p* = 0.0177; Figure 2D). Taking all these in account, BPIFB4 plasma levels are inversely correlated with disease severity, even though no significant correlations were found between BPIFB4 and other COVID-19 inflammatory and prognostic markers (CRP, D-dimer, ferritin, etc.; data not shown).

### Recombinant Human LAV-BPIFB4 Blunts Inflammatory Response to Lysates From SARS-CoV-2-infected Cells in PBMCs In Vitro

The reduced plasma level of BPIFB4 in COVID-19 patients led us to deeply investigate the putative protective role of BPIFB4 in in vitro studies.

LAV of BPIFB4 gene was described to protect from age-related endothelial dysfunction (21,22) and atherosclerosis, 2 main age-related conditions, mainly by conferring subjects with a favorable maintenance of nitric oxide bioavailability and huge anti-inflammatory profile. Thus, we explored the influence of recombinant LAV-BPIFB4 on the primary response of PBMCs from healthy donors upon stimulation with digested lysates from SARS-CoV-2-infected cells.

**Figure 3.**
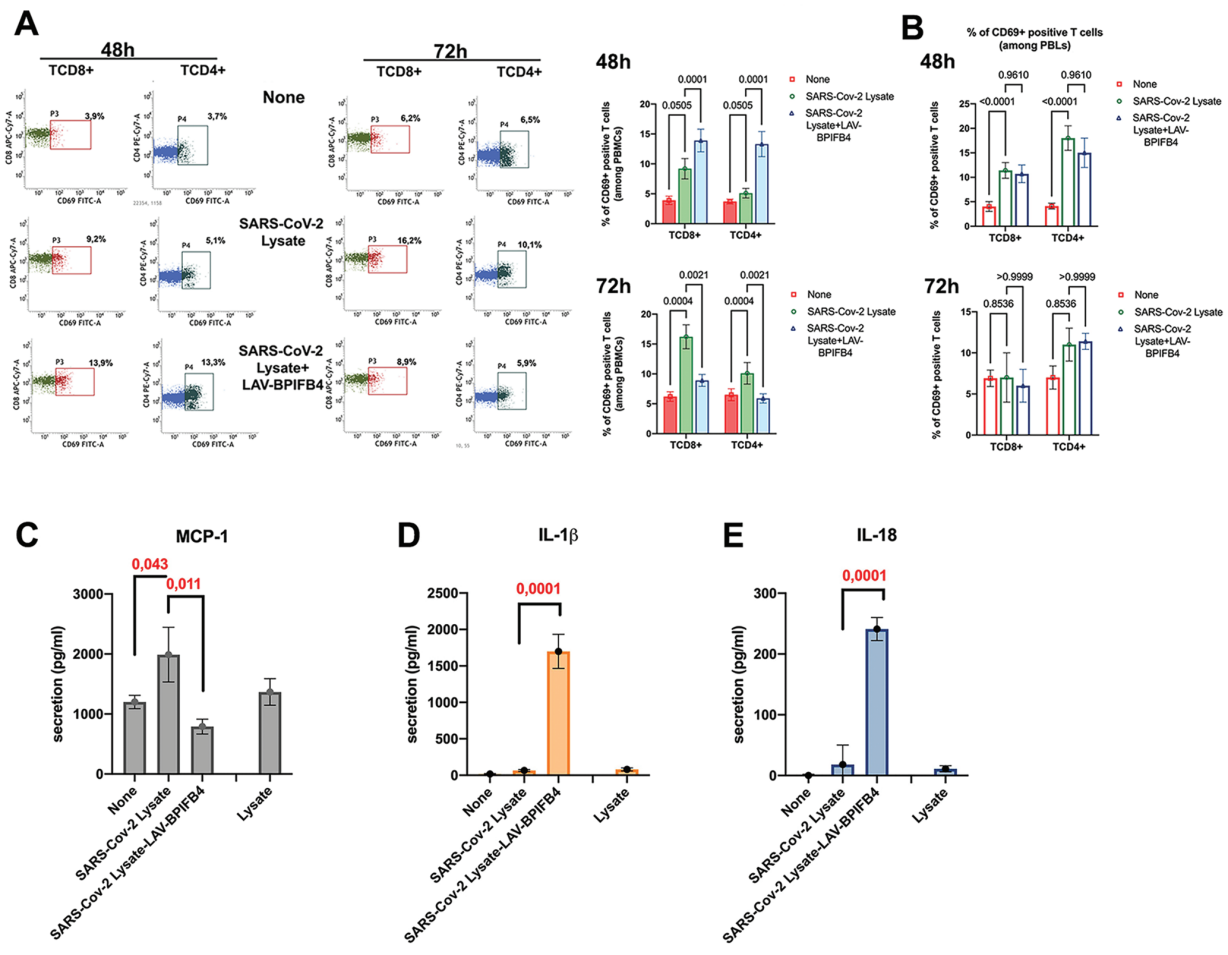
Recombinant human LAV-BPIFB4 tunes inflammatory response to lysates from SARS-CoV-2-infected cells in peripheral blood mononuclear cells (PBMCs) in vitro. After a 2-hour LAV-BPIFB4 pretreatment, healthy PBMCs were incubated with SARS-CoV-2 lysate (50 μg/mL) or control lysate for 48 hours and 72 hours. In order to avoid the “activation-induced cell death” (AICD) in vitro, after the first 2 hours, RPMI-FBS medium (with or without SARS-CoV-2 lysate) was discarded and fresh RPMI-FBS medium with rhLAV-BPIFB4 (18 ng/mL) was added for the following 46 hours or 70 hours. At the end of the cell culture, supernatants were collected and PBMCs assayed by FACS analysis after staining with anti-CD3, anti-CD4, anti-CD8, and anti-CD69 mAb. (**A**) A representative FACS dot plot is presented. Bar graphs report the percentage ± *SD* of CD69+ of both CD8+ gated TCD3+ cells and CD4+ gated TCD3+ cells from 3 independent experiments using different donors (analysis of variance [ANOVA] with correction for multiple comparisons using the Holm-Sidak method). (**B**) Bar graphs report the percentage ± SD of CD69+ of both CD8+ gated TCD3+ cells and CD4+ gated TCD3+ cells among peripheral blood lymphocytes obtained after CD14+ immunomagnetic depletion from PBMCs in 3 independent experiments using different donors (ANOVA with correction for multiple comparisons using the Holm-Sidak method). (**C–E**) Multiplex ELISA assay of cytokines’ release in medium after 72 hours of treatment. Results were expressed as the mean ± *SD* of all sample determinations conducted in triplicate. *p*-values indicate significance levels comparing average cytokines’ release among different groups (ANOVA).

In an experimental setting in vitro, we demonstrated an increased CD3+T cell reactivity (both TCD4+ and TCD8+) when healthy PBMCs were pulsed with SARS-CoV-2 lysates from 48 to 72 hours. Indeed, a significant higher percentage of CD69+ activated lymphocytes were found among SARS-Cov-2 lysates-treated PBMCs as compared to nontreated ones both at 48 hours (9.2 ± 1.7 vs 3.9 ± 0.7; *p = .*05 for TCD8+ cell subset and 5.1 ± 0.8 vs 3.7 ± 0.4; *p* = .05 for TCD4+ cell subset) and soon after at 72 hours (16.2 ± 2 vs 6.2 ± 0.8; *p* = .0004 for TCD8+ cell subset and 10.1 ± 1.8 vs. 6.5 ± 0.9; *p =* .0004 for TCD4+ cell subset). When PBMCs exposed to SARS-CoV-2 lysates were pretreated with rhLAV-BPIFB4, the expression of CD69 activation marker was modulated in a time-dependent manner. Specifically, at the early time points (48 hours after SARS-CoV2 lysate burst), the treatment with rhLAV-BPIFB4 induced an increase in the percentage of both CD69+TCD8+ (13.9 ± 1.9 vs. 9.2 ± 1.7; *p* = .0001) and CD69+TCD4+ (13.3 ± 2.1 vs 5.1 ± 0.8; *p* = .0001), establishing a primary line of defense. Later, 72 hours after SARS-CoV-2 lysate burst, rhLAV-BPIFB4 treatment significantly reduced the expression of CD69 activation marker on the surface of both TCD8+ (8.9 ± 1 vs 16.2 ± 2 vs. 6.2 ± 0.8; *p =* .0021) and TCD4+ (5.9 ± 0.78 vs. 10.1 ± 1.8; *p =* .0021; Figure 3A and B). Further, as most of the LAV-BPIFB4 immunomodulatory effects are related to myeloid compartment, we performed experiments of PBMCs activation in absence of CD14+ monocytes in order to verify if the latter are also determinants for LAV-BPIFB4 effect in response to SARS-CoV2 lysate stimulation. As expected, under the above conditions, ie the peripheral blood lymphocytes alone, rhLAV-BPIFB4 was not able to increase the percentage of both CD69+TCD4+ and CD69+TCD8+ at 48 hours, neither to reduce the expression of CD69+ activation marker on the surface of both T cell subsets later at 72 hours (Figure 3B). These results may point to a unique role of LAV-BPIFB4 in inducing a first protective response and then in reducing the magnitude of lymphocyte response upon persistent inflammatory stimuli. As consequence LAV-BPIFB4 treatment had also an effect on cytokine release in the virus lysate pulsed-PBMCs.

As shown in Figure 3C, a specific MCP-1 production was observed in PBMCs in response to stimulation by SARS CoV-2 lysate compared with control PBMCs pulsed with cell lysate alone (1989 ± 456 pg/mL/5 × 106 cells vs 1367 ± 221 pg/mL) or compared with nonpulsed PBMCs (1989 ± 456 pg/mL vs 1201 ± 112 pg/mL; *p =* .043). Consistent with its immunomodulatory role, we found that the pretreatment with rhLAV-BPIFB4 (18 ng/mL) significantly reduced MCP-1 release (790 ± 124 pg/mL vs 1989 ± 456 pg/mL; *p* = .011), which is responsible for abundant inflammatory cell infiltration to sites of infection in vivo.

It is well established that an imbalanced host immune response to SARS-CoV-2 drives COVID-19, as not only pro-inflammatory cues but also low innate antiviral defenses may contribute to disease development. Thus, we also tested the LAV-BPIFB4 effect on the secretion of the inflammasome-related cytokines IL-1β and IL-18, described as largely protective during murine coronavirus infection in vivo (23). Interestingly, SARS-CoV-2 lysate-pulsed PBMCs when preliminary exposed to LAV-BPIFB4, secreted significantly more IL-1β (Figure 3D) and IL-18 (Figure 3E) than non-pretreated PBMCs (1705 ± 234 vs 66 ± 12 pg/mL; *p* = .0001 for IL-1β and 241 ± 19 vs 18 ± 32 pg/mL; *p* = .0001 for IL-18), as determined by multiplex ELISA.

### LAV-BPIFB4 Pretreatment Protects Against SARS-CoV2 Cytotoxicity in Selected Target Cells

Concomitantly with an aberrant inflammatory response, SARS CoV-2 infection results in the damage of several target cells and viral dissemination in vivo.

To further validate the role of BPIFB4, whose level was found decreased in course of infection, we finally investigated its activity in cell damage of SARS-CoV-2 lysate against a panel of susceptible cell lines in vitro. We compared SARS-CoV-2-induced cytotoxicity in BEAS-2B (human bronchial epithelial cells), A549 (human alveolar basal epithelial cells), and HUVEC (human umbilical vein endothelial cells), in presence or absence of rhLAV-BPIFB4 (18 ng/mL).

As highlighted by the lactate dehydrogenase (LDH) release assay, a stable cytosolic enzyme that is released upon cell lysis, stimulation of target cells with SARS-CoV-2 lysate generated virus-specific cytolytic reactivities in vitro, as control lysate alone failed to induce cytotoxicity (80.2 ± 12.4 vs 15.3 ± 8% for BEAS-2B cells, 81.9 ± 9.4 vs 20.2 ± 4.5% for A549 cells, 30.1 ± 1.8 vs. 15.5 ± 3% for HUVEC cells).

**Figure 4.**
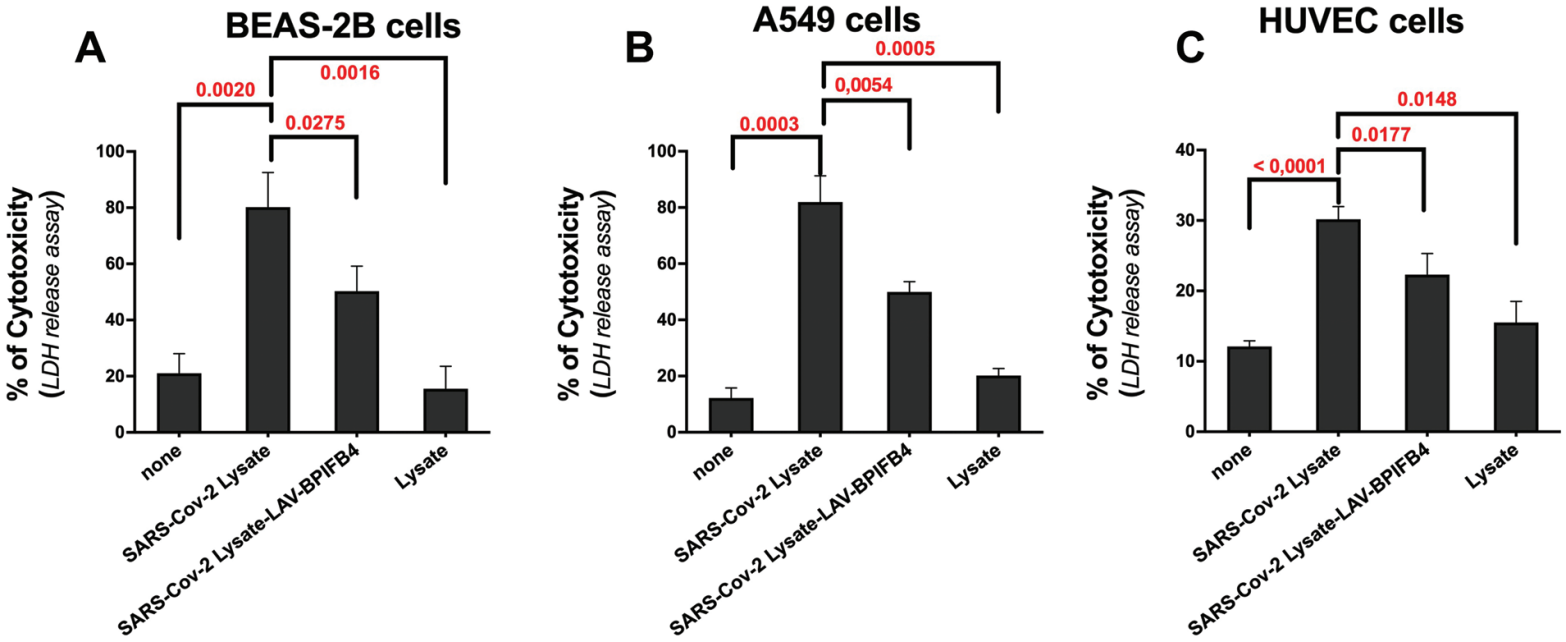
Protective effects of recombinant human LAV-BPIFB4 against SARS-CoV-2 lysate-induced cytotoxicity in vitro. Bar graph showing average cytotoxicity (±*SD*) determined using LDH assay in (**A**) BEAS-2B (human bronchial epithelial cells), (**B**) A549 (human alveolar basal epithelial cells), and (**C**) HUVEC (human umbilical vein endothelial cells) following 3 days treatment with SARS CoV-2 lysate or control lysate (50 μg/mL) in presence or absence of rhLAV-BPIFB4 (18 ng/mL; *n* = 3). *p*-values indicate significance levels comparing average lactate dehydrogenase release among different groups (analysis of variance).

The pretreatment with LAV-BPIFB4 significantly enhanced the cell viability and decreased LDH release in all target cells exposed to virus lysate versus non pretreated cells (50.3 ± 9 vs 80.2 ± 12.4% for BEAS-2B cells, 49.9 ± 3.7 vs 81.9 ± 9.4% for A549 cells, 21.8 ± 3 vs 30.1 ± 1.8% for HUVEC cells; Figure 4). These results suggested that circulating BPIFB4 levels in COVID-19 patients may limit the SARS-CoV-2 cell injury.

## Discussion

SARS-CoV-2, a member of the family Coronaviridae, has triggered a lethal pandemic termed coronavirus disease 2019 (COVID-19). A distinct characteristic of SARS-CoV-2 infection is the propensity to selectively induce morbidity and mortality in older people (24). The basis for the high lethality in older individuals is presumably connected to the *inflammaging* and a less robust immune system (7–9). Indeed, with time, a decline in immune efficacy, termed *immunesenescence*, and a deleterious secretory phenotype of senescent cells (SASP) generate that low-grade inflammatory background which may exacerbate the COVID-19 vulnerability of the older adults and its cardiovascular implications of fatal outcomes.

LLIs, by maintaining a fine balance between inflammatory and anti-inflammatory circuits, age slowly and healthy, avoiding chronic cardiovascular diseases. Accordingly, while mortality increases up to very old ages, a resilience of men older than 90 years was documented in the North of Italy (12). Furthermore, clinical and epidemiological data from Cilento, a rural area in the Southern of Italy mainly enriched of nonagenarians and centenarians, indicated that LLIs develop more resiliency to COVID-19, in term both of the occurrence of pathological event and a better tendency to illness’ recovery (personal communication).

In this scenario, the immune asset and soluble factors characterizing the long lifespan of LLIs may offer new diagnostic and therapeutic opportunities in COVID-19 pandemic.

The LAV of the bactericidal/permeability-increasing fold-containing-family-B-member-4 (BPIFB4) has been associated with exceptional longevity, and gene therapy with this isoform was able to improve revascularization and endothelial function (21). Furthermore, atherosclerotic process was blunted by LAV-BPIFB4 in a mouse and ex vivo human model (18); at the same way, diabetic cardiopathy was attenuated by LAV-BPIFB4 gene transfer in mice model of disease (25). These effects can be in part explained by the LAV-BPIFB4 ability to influence the polarization of human and murine monocytes by tuning their differentiation process toward dendritic cells with regulatory functions (IL-10 and TGF-beta producing cells) in human healthy donors (14) and toward pro-resolving M2 macrophages in ApoE-/- mice (18), also by positively affecting the SDF-1/CXCR4 axis (18,25). To be noted, BPIFB4 is a secreted protein belonging to the BPI/lipopolysaccharide-binding protein (LBP) family of antibacterial components that participates in host protection through antimicrobial and surfactant properties at the upper airways (13). A recent study showed that the ACE2-enriched mucosa of oral cavity can be responsible for the virus easy access to a new susceptible host (26) and underpins the importance of full competent cellular and humoral artillery to cope with the virus spreading.

We have recently described that BPIFB4 levels are increased in plasma and PBMCs of LLIs (15,27), in CD34+ cells of LLIs (21) and in serum of healthy versus non-healthy (frail) LLIs (16) and are closely related to a balanced immune response both dampening the deleterious activation and counterbalancing the immune decline in steady state.

The hypothesis is that BPIFB4, originally belonging to a family of host defense proteins, (28), may be a new longevity-associated determinant of COVID-19 patients. Our observational clinical study on *n* = 64 COVID-19 patients helped to assess the predictive value of blood level of BPIFB4 of COVID-19.

Indeed the reduced circulating levels of BPIFB4 in patients with severe disease (characterized by elevated levels of CRP and LDH) validate its prognostic significance for severity in patients with COVID-19 (Figures 1 and 2). This is in agreement with previous findings by our group highlighting the importance of BPIFB4 levels to classify both the health status of LLIs (by discriminating frail individuals vs non-frail ones) (16) and to predict the atherosclerotic risk being the protein’s concentration significantly higher in subclinical carotid atherosclerosis and in patients with IMT <2 mm, as compared to patients with carotid stenosis (18).

Of note, the lack of correlations between circulating concentrations of BPIFB4 and other inflammatory markers (CRP, LDH, ferritin, D-dimer, lymphocyte count, etc., data not shown) may be consistent with the hypothesis that the low levels of BPIFB4 in high-grade COVID-19 patients may represent at most the cause and not the consequence of the disease. It is unlikely that low level of BPIFB4 in high severity group may be related to its consumption by the high inflammatory background; rather we hypothesize that based on genotype, or the peculiar stability of the protein, the high circulating level of BPIFB4 characterizing some COVID-19 patients, may induce a homeostatic response and a better tendency to counteract an inflammatory background as seen in the context of “inflammaging” of old individuals. Indeed, as LLIs that carry high levels of BPIFB4, are able to better cope with disabilities linked to inflammatory conditions, at the same way high level of BPIFB4 can blunt the inflammatory burst typical of the COVID-19.

The functional role of the circulating BPIFB4 was magnified when examining its contribution to properly tune the inflammatory response of healthy PBMCs to SARS-CoV-2 lysate (Figure 3). In a monocyte-dependent manner, LAV-BPIFB4 was able to induce a distinct T cell response upon SARS-CoV-2 lysate stimulation, described to be both protective (48 hours) and self-limiting (72 hours; Figure 3A and B), that would be relevant in conferring protection from the “cytokine storm” to which several tissues, such as bronchial cells (Supplementary Figure 1B), are exposed in COVID-19.

Indeed, in a second experimental setting in vitro, we confirmed the protective role of high protein levels of BPIFB4 by decreasing MCP-1 responsible for hyper-innate inflammatory response in SARS-CoV-2 infection through the recruitment of mono/macrophages and neutrophils in vivo (29). Again, the finding is in line with the observed reductions of CD68-positive cells at the aortic arch level and of MOMA-2 mono-macrophage-positive cells in femoral arteries of ApoE knockout mice after systemic LAV-BPIFB4 gene therapy using an adeno-associated viral vector (18). The well-established effect of BPIFB4 protein on the mono-macrophage compartment was also highlighted by the peculiar redistribution of monocyte pool, which uniquely marks LLIs. As documented elsewhere, circulating levels of nonclassical CD14+CD16+ *patrolling monocyte* (PMo) were found significantly higher in LLIs compared to young and old controls (27). Patrolling monocytes actively patrol the resting vasculature to remove injured cells in a number of chronic conditions where they have been described to assist wound healing process and the resolution of inflammation (30). This functional association together with the degree of protection from hypertension, ischemia and atherosclerosis offered by LAV-BPIFB4 treatment, could in part explain the reduced occurrence of chronic cardiovascular diseases in LLIs, endowing them also with the capacity to better recover after SARS-CoV-2 infection, too.

This is confirmed by the ability of rhLAV-BPIFB4 to limit cellular damage in vitro after SARS-CoV-2 lysate (Figure 4) probably mediating a cellular homeostatic response to stress, as already documented for STHdh Q111/111 striatal cells exposed to cytotoxic insult of an expanded CAG repeats encoding a long polyglutamine tract in the huntingtin protein (Htt) (31). Indeed, a stress response process was facilitated by the LAV-variant consisting in BPIFB4 phosphorylation/activation by stress kinase protein kinase R-like endoplasmic reticulum kinase-PERK, its complexing with 14-3-3 and HSP 90 and calcium/endothelial nitric oxide synthase activation (20–22). The NLRP3 inflammasomes, a complex of multiple proteins that activates the secretion of the proinflammatory cytokine (eg, IL-1β and IL-18) in a caspase-1-dependent manner, often participate in the regulation of an optimal endoplasmic reticulum stress response (ERS) in the injured cell. However, while a transient protective ERS restores protein homeostasis by activating the UPR and reducing protein aggregates, long-term or severe ERS can trigger cell dysfunction and death (32,33). In our opinion, this protective response mediated by LAV-BPIFB4 may also explain the enhancing production of IL-1β (Figure 3D) and IL-18 (Figure 3E), well-known inflammatory cytokines with a variety of immunomodulatory effects, able of leading to both early host protection and damaging pathological events after chronic deleterious perpetuation of inflammatory response. Among protective effects, IL-18 signaling promotes production of interferon gamma and perforin-mediated cytotoxicity (34–36), crucial for viral clearance. At the same way, IL-1 signaling controls viral replication and the induction of a protective T cell response during virus infection (37,38).

Interestingly, IL-18 was found to be important for host defense, as suggested by the poor survival and elevated viral replication in mice lacking IL-18 during murine coronavirus infection (23).

Even though the cellular mechanism and the regulatory circuits by which LAV-BPIFB4 induces a homeostatic response are still unknown, the relevance covered by the biphasic immune responses (protective and self-limiting) and the fine tuning of cytokines milieu might in part explain the beneficial effect achieved by LAV-BPIFB4 in the context of COVID-19.

In conclusion, even if the associative nature of data does not permit to definitively conclude that the BPIFB4 plasma level is relevant to the COVID-19 prognosis, to better of our knowledge, our present work constitutes the first study to describe a longevity-associated protein discriminating among severity-based stratified COVID-19 patients.

From a translational point of view, a better characterization of BPIFB4 clinical relevance by investigating its expression in damaged tissues or PBMCs obtained from COVID-19 patients will help to validate BPIFB4 as a valuable biomarker for COVID-19 severity. This may be useful in a more accurate stratification of patients, their management and in treatment decision. Furthermore, its dual role on immune compartment and in fruitful stress response to limit cellular damage makes BPIFB4 an attractive therapeutic tool to counteract complications of COVID-19.

## Supplementary Material

Supplementary data are available at *The Journals of Gerontology, Series A: Biological Sciences and Medical Sciences* online.

Supplementary Figure 1. Cytokine report analysis conducted by multiplex ELISA on the supernatants of BEAS-2B and HUVEC cells after 72 hours of treatment with 50 μg/mL of SARS-CoV-2 Lysate in presence or absence of 18 ng/mL of rhLAV-BPIFB4 (**A**). In the experiment shown in the lower panel, BEAS-2B cells were stimulated for 72 hours with conditioned medium CM (diluted 1/5) from resting PBMCs, SARS-CoV-2 treated-PBMCs or LAV-BPIFB4-SARS-CoV-2 co-treated PBMCs. Bar graph showing average cytotoxicity (±SD) determined using LDH assay. P-values indicate significance levels comparing average LDH release among different groups (ANOVA). The data clearly suggest that the positive modulation of PBMCs-cytokine milieu by rhLAV-BPIFB4 would confer protection of bronchial cells during the inflammatory response induced by SARS-CoV-2 (**B**).

glab208_suppl_Supplementary_Material
