# Supplementary material for: BPIFB4 Circulating Levels and Its Prognostic Relevance in COVID-19
Source: J Gerontol A Biol Sci Med Sci. 2021 Aug 16;76(10):1775–83. doi: 10.1093/gerona/glab208 (PMC8436991; doi:10.1093/gerona/glab208)
Supplement: glab208_suppl_Supplementary_Material [file glab208_suppl_supplementary_material.pdf]

BEAS-2B

Name	Dilution	MCP-1	IL-6	TNF-α	IL-1β	IL-8	IL-10	IL-1beta	INF-alpha2	IFN-gamma	IL-12p70	IL-17a	IL-18	IL-23	IL-33
		pg/ml	pg/ml	pg/ml	pg/ml	pg/ml	pg/ml	pg/ml	pg/ml			pg/ml	pg/ml	pg/ml	
BEAS-2B alone	1	30,00	28,74	2,91	<2.27	557,64	<2.24	<2.27	<2.28	<2.29	<2.30	<2.31	<2.32	<2.33	<2.34
BEAS-2B SARS-CoV2Lysate	1	34,67	291,06	<2,38	<2.27	2708,59	<2.24	<2.27	<2.28	<2.29	<2.30	<2.31	<2.32	<2.33	<2.34
BEAS-2B SARS-CoV2Lysate+LAV-BPIFB4	1	35,56	280,43	<2,38	<2.27	3129,39	<2.24	<2.27	<2.28	<2.29	<2.30	<2.31	<2.32	<2.33	<2.34

HUVEC

Name	Dilution	MCP-1	IL-6	TNF-α	IL-1β	IL-8	IL-10	IL-1beta	INF-alpha2	IFN-gamma	IL-12p70	IL-17a	IL-18	IL-23	IL-33
		pg/ml	pg/ml	pg/ml	pg/ml	pg/ml	pg/ml	pg/ml	pg/ml			pg/ml	pg/ml	pg/ml	
HUVEC alone	1	1419.89	2630,49	25,45	2,71	>12350,74	14,95	<2.27	<2.28	<2.29	<2.30	<2.31	<2.32	<2.33	<2.34
HUVEC SARS-CoV2Lysate		1338,54	2548,20	5,92	2,71	>12350,74	14,95	<2.27	<2.28	<2.29	<2.30	<2.31	<2.32	<2.33	<2.34
HUVEC SARS-CoV2Lysate +LAV-BPIFB4		875,12	1519,82	25,45	2,71	>12350,74	14,95	<2.27	<2.28	<2.29	<2.30	<2.31	<2.32	<2.33	<2.34

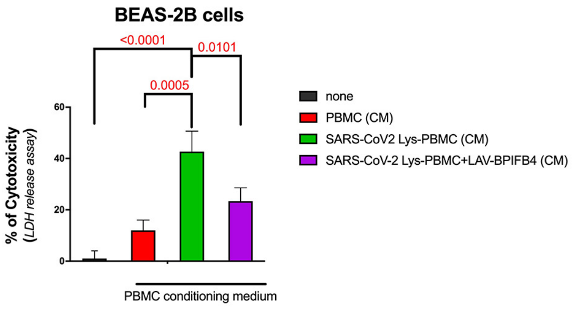

Supplementary Figure 1

Supplementary Table 1

Laboratory Analysis	Mean ± SD; Median
**CPR** (mg/dl; normal range 0-4,5)	5,81 ± 8,76; 2,1
**D-Dimer** (ng/ml; normal range <500)	951,19 ± 1715,30; 440
**Erythrocyte Sedimentation Rate (mm/hr,** normal range 0-22**)**	41,06 ± 24,59; 35
**Ferritin** (ug/L; normal range 15-200)	467,10 ± 464,80; 388,6
**Fibrinogen** (mg/dl; normal range 150-400)	431,27 ± 217,42; 408,5
**Hematocrit** (%; normal range 35-48 %)	37,62 ± 5,57; 37,7
**Hemoglobin** (d/dl; normal range 12-15 g/dl)	12,21 ± 2,02; 12
**LDH** (U/L; normal range 0-252)	415,94 ± 287,54; 338
**Leucocytes** (10^3/µL; normal range 4,5-11)	8,06 ± 4,09; 6,6
**Lymphocytes** (10^3/µL; normal range 1,0-4,8)	1,53 ± 0,80; 1,51
**Mean Corpuscular Volume** (fL; normal range 80-96 fL)	89,08 ± 7,76; 89
**Platelet** (10^3/µL, normal range 150-450)	227,76 ± 104,98; 214,5
